# Public Health at the Anglo-Japanese Exhibition

**Published:** 1910-01-29

**Authors:** H. D. Searles-Wood

**Affiliations:** Chairman, Public Health and Sanitation Section, Japan-British Exhibition


### PUBLIC HEALTH AT THE ANGLO-JAPANESE EXHIBITION.

*To the Editor of* The Hospital.

Sir,—The Japanese exhibits under the auspices of the Imperial Japanese Government, at the Japan-British Exhibition at the White City next summer, promise to be a complete demonstration of the progress made by that nation. It is absolutely necessary, therefore, if we as a nation are to be adequately represented, that immediate steps be taken to render the British exhibits an equal demonstration of the progress made by the United Kingdom. As President of the Section of Public Health and Sanitation I hope you will allow me to call the attention of all those who are engaged in matters which come within the scope of this section to help in making it worthy of the occasion.

Great Britain is the leader in all matters connected with hygiene, and it is hoped that this section will adequately exhibit all that our most advanced sanitarians are doing. The Japanese Government and people are keenly interested in all matters connected with public health and sanitation, and already are the purchasers of our sanitary goods, and are anxious to know where the most perfect appliances can be obtained.

It is with a view of calling upon the patriotism of sanitarians to see that this section worthily represents the place that Great Britain holds in sanitary science that I venture to make this appeal to them.

Yours faithfully,
H. D. Searles-Wood,
Chairman, Public Health and Sanitation Section,
Japan-British Exhibition.
January 25, 1910.

